# The Eclectic Nature of Glioma-Infiltrating Macrophages and Microglia

**DOI:** 10.3390/ijms222413382

**Published:** 2021-12-13

**Authors:** Víctor A. Arrieta, Hinda Najem, Edgar Petrosyan, Catalina Lee-Chang, Peiwen Chen, Adam M. Sonabend, Amy B. Heimberger

**Affiliations:** 1 Department of Neurosurgery, Lou and Jean Malnati Brain Tumor Institute, Robert H Lurie Comprehensive Cancer Center, Northwestern University Feinberg School of Medicine, Chicago, IL 60611, USA; victorarrieta@northwestern.edu (V.A.A.); hinda.najem@northwestern.edu (H.N.); edgar.petrosyan@northwestern.edu (E.P.); catalina.leechang@northwestern.edu (C.L.-C.); peiwen.chen@northwestern.edu (P.C.); 2 PECEM, Facultad de Medicina, Universidad Nacional Autónoma de México, Mexico City 04360, Mexico

**Keywords:** microglia, macrophages, gliomas, immunotherapy

## Abstract

Glioblastomas (GBMs) are complex ecosystems composed of highly multifaceted tumor and myeloid cells capable of responding to different environmental pressures, including therapies. Recent studies have uncovered the diverse phenotypical identities of brain-populating myeloid cells. Differences in the immune proportions and phenotypes within tumors seem to be dictated by molecular features of glioma cells. Furthermore, increasing evidence underscores the significance of interactions between myeloid cells and glioma cells that allow them to evolve in a synergistic fashion to sustain tumor growth. In this review, we revisit the current understanding of glioma-infiltrating myeloid cells and their dialogue with tumor cells in consideration of their increasing recognition in response and resistance to immunotherapies as well as the immune impact of the current chemoradiotherapy used to treat gliomas.

## 1. Introduction

The idea of the central nervous system (CNS) being immunologically privileged was based on the misconception of an impenetrable and quiescent state. However, this long-standing concept has been challenged and refuted. This change in perspective regarding CNS immune reactivity derives in part from the findings of an unrecognized meningeal lymphatic vascular system in the CNS [1,2,3]. This has contributed to the understanding of the means that are used by the immune cells and macromolecules to be transported outside the brain. Additionally, contiguous communication between the skull bone marrow and the brain provides an avenue for active traffic of myeloid cells to respond under emerging disturbances such as brain tumors, to CNS homeostasis [4]. Brain tumors release signals into the cerebrospinal fluid which communicates with the skull bone marrow to instruct cranial hematopoiesis [5]. This recent evidence further underscores the dynamic and unique nature of the brain immune system.

Myeloid cells, constituting microglia and monocytes that give rise to macrophages and dendritic cells, conglomerate in gliomas to exert a number of simultaneous actions in defending or supporting tumor growth. The convergence of different myeloid cells from diverse hematopoietic organs in gliomas likely contributes to the tumor cell heterogeneity described in these brain tumors. Estimations consider that around 30–50% of the tumor content is represented by monocyte-derived macrophages (MDMs) and resident microglia [6,7]. The mechanisms used by MDMs and microglia to populate gliomas are influenced by their embryological origins, spatial localization, and functional nature. As part of the innate immune defense, myeloid cells engulf tumor components, present antigens, produce cytokines, and induce direct cytotoxic activities on damaged cells, pathogens, and tumor cells [8,9].

Single-cell technologies have enabled exquisite characterizations of the multidimensional phenotypic states of the myeloid cells in different brain geographic regions, in age, in health, and in the context of neurological diseases [10,11,12,13], including gliomas [14,15,16,17,18]. Glioma-infiltrating myeloid cells show a wide range of transcriptional phenotypes derived from the presence of both MDMs and microglia [14,15,16]. Despite this evidence, many studies consider macrophages and microglia as single myeloid entities. Instead, the recognition of microglia and MDMs as different entities as well as the heterogeneity of each of these immune cell populations in the context of brain tumors will clarify the mechanisms of response and resistance to immunotherapies attributed to these cells.

Interactions and spatial relationships between cancer and immune cells were shown to be associated with clinical responses to immunotherapies in some cancers [19,20,21]. In gliomas, the interaction between cancer cells and immune cells was shown to guide tumors in specific evolutionary transcriptional trajectories [22]. Thus, it is highly plausible that glioma and immune cell interactions determine tumor immunogenicity and clinical responses to immunotherapy in patients where this cellular dialogue is more active. In this review, we provide a perspective of the role of each of the myeloid cells under the influence of gliomas and the impact of therapies on these immune cells.

## 2. Dissecting Myeloid Cells in Gliomas: Multiple Actors Come into Play

Several differences between microglia and MDMs have been described comprehensively in gliomas and other neurological diseases. Particularly, microglia are immune cells derived from primitive yolk sac macrophages that arise during early embryogenesis [23,24]. This brain-specific type of cell was preserved through the evolution of species to maintain CNS homeostasis, shape the neuronal networks, and cope with pathogens and insults of different origins. Though some microglia display remarkable longevity [25], the pool of these immune cells is sustained by modest local expansion throughout adult life with different proliferative rates depending on the brain region [26]. During neuropathological conditions, specific microglial clones expand to contend with CNS damage and subsequently decrease in cell number upon returning to homeostatic states [26]. Although little is known about the proliferation dynamics of microglia under the influence of gliomas, microglia and MDM from gliomas possess the proliferating capacity that was assessed by Ki-67 expression [27,28]. This suggests that like other neurological diseases, microglial cells respond to oncogenic insults through proliferation and phenotypic changes.

**Figure 1:**
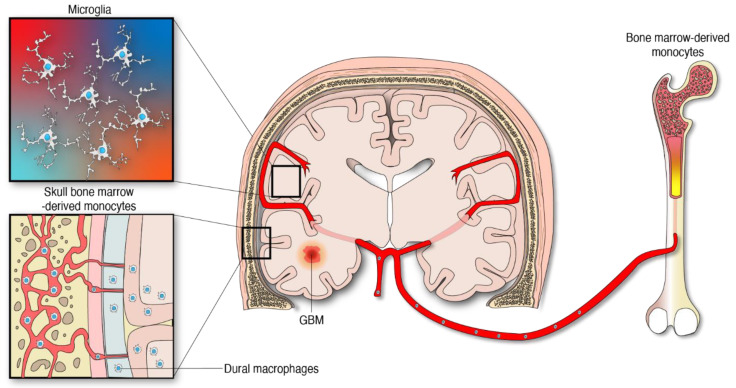
Myeloid cells from different anatomical and embryological origins contribute to the cellular heterogeneity in gliomas.

Apart from the microglia, specialized subsets of macrophages exist in the CNS borders including the perivascular, leptomeningeal, choroid plexus, and dural niches [29]. CNS-associated macrophages are diverse and long-lived. Some of these cell populations are replenished via local self-renewal and others by bone-marrow macrophages [30,31]. The modulation and influence of these macrophages by gliomas is an area of increasing investigation. The other abundant macrophage population infiltrating gliomas emanate from peripheral monocytes which are generated from hematopoietic progenitors in the bone marrow [6], Figure 1. Importantly, the skull and the vertebral bone marrow are prominent sources of monocytes that travel through vascular channels to settle in the meninges and the brain parenchyma under homeostatic and neuroinflammatory conditions [4,32]. Considering this new evidence, it is reasonable to infer that monocyte progenitors derived from skull bone marrow contribute to the supply of MDMs in the tumor microenvironment (TME) of gliomas. However, the extent of this contribution has not yet been defined. The peripheral immune compartment of glioma patients is also a source of tumor-infiltrating myeloid cells. Particularly, relevant numbers of myeloid-derived suppressor cells (MDSCs) were found in matched peripheral blood and tumors of patients with GBM [33,34], the most aggressive type of gliomas classified as WHO grade 4 tumors [35]. MDSCs are myeloid progenitors at earlier stages of differentiation that develop and accumulate systemically and in the TME where they arrive to promote an immunosuppressive milieu in support of gliomagenesis [36,37].

Recently, it was shown that cancer-driven emergency myelopoiesis, an increase in myeloid cells derived from cancer-related inflammation, leads to an increase in MDSCs in the periphery [38]. During this process, PD-1 and PD-L1 are expressed by myeloid cell progenitors such as monocytic MDSCs and polymorphonuclear MDSCs. PD-1 is an immune checkpoint receptor expressed by several immune cells including T cells, B cells, NK cells and myeloid cells, that binds to PD-L1/2 to reduce immune responses [39]. Interestingly, the *Pdcd1* deletion moved the myeloid progenitors away from the immunosuppressive MDSCs phenotype by increasing the expression of *Irf8*. This highlights that the immunosuppressive phenotype of myeloid cells may be acquired peripherally, at least in part, during the process of cancer-driven emergency myelopoiesis with potential modulation by immune checkpoint inhibitors. Of course, TME-induced immune suppression has been extensively described. Even though MDSCs are known to infiltrate the TME of GBM [33], the process of cancer-driven emergency myelopoiesis has not been investigated in these brain tumors. Notably, in murine models of high-grade gliomas, systemic administration of anti-PD-1 therapy triggered phenotypic changes of the myeloid cells and microglia within the TME indicating that: (1) biomarkers of response need to include the innate immune compartment; and (2) that there may be important immune-modulatory roles within the TME mediated by these drugs [40].

The specificities of the genetic profile of each glioma can influence the capabilities of recruiting MDMs. An example of this phenomenon is the recent evidence showing that *PTEN* loss of function in glioma cells induces the secretion of lysyl oxidase which recruits MDMs to the tumor [41]. Additionally, the combined activity between epidermal growth factor receptor (EGFR) and the EGFRvIII in glioma cells induces signaling through KRAS to increase the expression of CCL2 [42], a ligand that binds with high affinity to the CCR2 receptor expressed by monocytes derived from the peripheral blood and the skull bone marrow [4,43]. Additionally, multiple chemokines are secreted by glioma cells that play key roles in MDM migration into the TME [43,44,45,46,47,48,49].

Collectively, gliomas incite their colonization by immune cells coming from local, contiguous, and peripheral sources through the induction of a local inflammatory process in response to the ongoing tumoral progression or the release of cancer cellular factors. Furthermore, the molecular characterization of each tumor has the potential to influence the degree of immune cell infiltration.

## 3. Differentiation Trajectories of Myeloid Cells under the Influence of Gliomas

The variability in immune activation and suppressive components among gliomas depends on the subtype and their localization in the brain [17,50]. In addition to these factors, newly diagnosed and recurrent GBMs differ in their immune composition and diversity due to the natural course of tumor progression and chemoradiotherapy [18]. Infiltration of peripheral monocyte/macrophages occurs in the early phases of tumor development and is maintained by constant immune cell recruitment throughout tumor progression as shown in murine glioma models [51,52]. This phenomenon is reflected in human gliomas where there are higher expression levels of MDMs genes compared to microglial genes in advanced stages of brain tumor disease irrespective of *IDH* mutational status [14,53]. From a different angle, microglia comprise the major proportion of myeloid cells in newly diagnosed GBM; whereas MDMs represent the most abundant myeloid cell population in recurrent GBMs [18]. The shift in the predominance of the myeloid cell population from microglia to MDMs suggests that these immune cells may compete for space in gliomas. Indeed, it was documented that the blockade of monocyte tumor infiltration results in a consequential increase in microglial cell numbers [18,54]. The spatial distribution of MDMs and microglia is represented in patches and interspersed between glioma cells throughout the entire tumor. These observations contrast with the notion of microglia being predominantly present at the tumor margin, a phenomenon seen in transplantable mouse glioma models [18]. Despite indicators of competition between microglia and MDMs in gliomas, many more immune cell populations are recruited and coexist in the complex ecosystem of gliomas.

**Figure 2:**
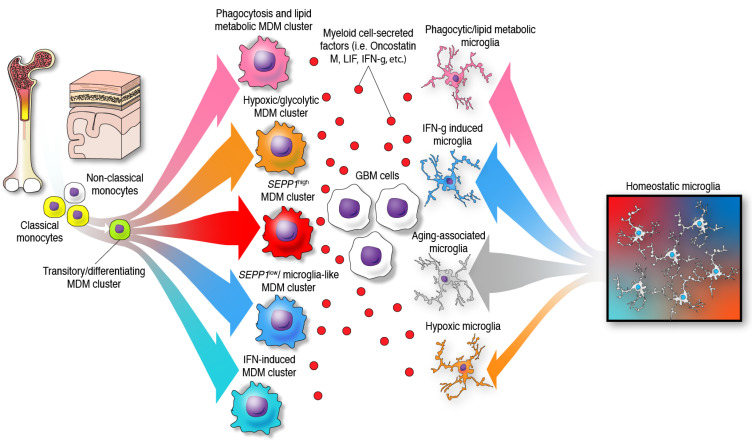
Identity and differentiation trajectories of MDMs and microglia under the influence of GBM cells in the TME. In the presence of gliomas, monocytes and microglia undergo differentiation towards different specific phenotypes.

The previous paradigm of categorizing glioma-infiltrating myeloid cells into M1 (anti-tumor, pro-inflammatory) and M2 (pro-tumor, immunosuppressive), whereas simple and convenient, does not represent the phenotypic diversity of these myeloid cells in the brain. Furthermore, the application of a nomenclature derived from in vitro functional characterization has led to a misunderstanding of the specific functions of glioma-infiltrating myeloid cells that is only now beginning to be clarified with single-cell sequencing. The multifaceted transcriptional phenotypes of myeloid cells uncovered in recent studies surpass the conventional M1/M2 classification as demonstrated by the following observations: a diverse expression of phenotypical states, mutually exclusive transcriptional programs when comparing glioma-infiltrating macrophages, lack of separation among these activated/alternative states, and co-expression of M1 and M2 markers such as IFN-γ and IL-4, among other markers by these immune cells [22,27]. Vast and compelling evidence arguing that the M1/M2 macrophage classification should be reconsidered was eloquently reviewed elsewhere [55]. Most importantly, this nomenclature appears misleading in terms of the semantics and the understanding of the cellular myeloid mosaic in GBM. Instead, the notion of multidimensional states of macrophages was suggested considering the spectrum of phenotypes displayed by macrophages under a wide variety of stimuli [56]. In the context of human gliomas, this phenomenon is illustrated by bioinformatic inferences of differentiation trajectories using single-cell technologies. As an example, the process of monocyte to macrophage transition was delineated by proteomic fate mapping in *IDH* wild-type and mutant gliomas [53]. This analysis showed three phenotype trajectories that monocytes follow upon invasion of glioma tissues. Single-cell RNA-seq analysis of GBM-infiltrating MDMs revealed a variety of macrophage clusters including: (1) a monocyte–macrophage transition; (2) phagocytic active/high lipid metabolism; (3) hypoxic/glycolysis active; (4) *SEPP1*^low^/ microglia-like; (5) *SEPP1*^high^/anti-inflammatory cluster and a (6) IFN-induced signature cluster [18]. The existence of these clusters suggests that upon invasion of glioma tissues, monocytes differentiate into macrophages and acquire specialized functions. In contrast with the idea of a continuum between two phenotypes, this further highlights the multiplicity of possibilities in which these leukocytes can transform in the context of brain malignancy, Figure 2. Furthermore, these analyses show multiple myeloid cell functions occurring simultaneously in the TME of gliomas.

Regarding the wide variety of immune functions of glioma-infiltrating myeloid cells, several immunosuppressive and tumor-supporting effects were described for these immune cells. Particularly, survival, maintenance, and phenotype education of MDMs towards a tumor-supportive phenotype are mediated by the secretion of glioma-derived factors, including CSF-1, periostin, and NO [44,57,58]. Once in the tumor site, MDMs produce anti-inflammatory cytokines such as IL-10 [59], transforming growth factor-beta (TGF-β) [60], IL-6 [61], pleiotrophin [62], as well as molecules with tissue remodeling and angiogenesis properties such as VEGF [63], MMP2 [64], MMP9 [65] and versican-induced MT1-MMP [66]. The interaction between MDMs and glioma cells results in increased invasion and tumor growth, which is facilitated by immunosuppression of the TME [6,67]. For instance, the phenotype of myeloid cells isolated from *IDH* wild-type GBM appears to be related to angiogenesis, proteolytic activity, and presence of growth factors such as PDGFA, TGFBI, SPP1 and GDF15, which explains their prevalence in perivascular and hypoxic regions.

A recent study showed that the scavenger receptor MARCO, expressed by MDMs existing predominantly in *IDH* wild-type GBMs, was associated with a mesenchymal profile and worse prognosis [28,68]. Gene set enrichment analysis revealed upregulation of gene signatures related to epithelial–mesenchymal transition, glycolysis, angiogenesis, and hypoxia in MARCO-expressing macrophages localized in the tumor core. Similar gene signatures related to remodeling of the TME were enriched in MDMs-infiltrating gliomas [27]. Additionally, proinflammatory gene signatures related to IFN-α, IFN-γ, and TNF-α signaling via NF-κB were downregulated in *MARCO*-expressing macrophages [28]. It is likely that other drivers such as CXCR4 will be identified that contribute to the mesenchymal transition [69].

Another example of the uniqueness of the GBM TME is the existence of a particular subpopulation of CD68^+^ macrophages expressing CD73 [70]. These CD73^+^ macrophages have a gene expression profile suggestive of MDMs whose phenotype involves a highly immunosuppressive and hypoxic gene signature similar to the one displayed by MARCO-expressing macrophages. CD39 and CD73 are part of an immunosuppressive pathway that converts ATP to adenosine which binds to the A2a receptor (A2aR) on effector T cells rendering them dysfunctional [71]. Indeed, the A2aR/CD39/CD73 adenosine pathway was found to be ubiquitous among other immune-related pathways in glioma-infiltrating immune cells such as CD11b^+^ cells and T cells. In addition, glioma-infiltrating myeloid and T cells exhibit higher expression of A2aR/CD39/CD73 adenosine pathway compared to GBM patient-matched PBMCs and healthy donor PBMCs [72].

MDSCs blood levels are high in glioma patients and show an increase as the grade of malignancy increases [34,73]. MDSCs promote immune escape by suppressing T cell proliferation and anti-tumoral responses in gliomas [36]. One of the suppressive T cell mechanisms of MDSCs is increased arginase activity detected in the serum and tumor tissue of glioma patients [73,74,75]. Arginase decreases L-arginine levels which is critical for the re-expression of the T cell co-receptor CD3ζ which sustains T cell activation and proliferation [76]. In this regard, GM-CSF, which is expressed at high levels in human gliomas, induces the upregulation of IL-4Rα on glioma-infiltrating MDSCs, thereby leading to IL-13-induced production of arginase resulting in T cell inhibition [77]. MDSCs also modulate T cell cytokine production as seen by suppressed IFN-γ T cell production [73,74].

Because of these immunosuppressive features of MDMs, strategies to deplete or modulate these immune cells were attempted in clinical trials. For instance, CSF-1R inhibitors were investigated, albeit they did not lead to better survival outcomes in GBM patients [78]. One of the explanations for this lack of efficacy is the acquisition of resistance to CSF-1R inhibitors induced by aberrant activation of the phosphatidylinositol 3-kinase (PI3K) pathway driven by paracrine signaling between macrophages and tumor cells [79].

Microglia also develop specialized functions upon interaction and stimulation with internal and external agents. Similar to neuroinflammatory diseases, the transformation of microglia also occurs under the influence of gliomas, leading to a downregulation of homeostatic core genes while upregulating disease-associated microglial genes [17]. Particularly, unique microglial clusters were identified in the context of GBM characterized by expressing interferon and hypoxia/angiogenesis-related genes [17,18]. Furthermore, microglia expressing phagocytic/lipid signatures are found in newly diagnosed and recurrent GBMs [18]. While a microglia hypoxic signature was identified in GBMs, the proportion of these microglial clusters was substantially small in comparison to the hypoxic MDM cluster [18]. Furthermore, this microglia cluster was found exclusively in recurrent tumors [18]. Interestingly, several of the microglial clusters within GBM were enriched in the gene ontology term “antigen processing via MHC Class I” and some had higher expression of HL-DR compared to microglial cells from control individuals. Such observations suggest that microglia have the potential to present antigens to T cells [17].

Inferences of phenotype trajectories revealed a sequence starting from homeostatic microglia acquiring an age-associated phenotype and ending as GBM-associated microglia. The modeling of this sequential transcriptional change in microglia was characterized by downregulation of microglial signature core genes such as *P2RY12*, *CXCR1*, *SELPGL*, *CSF1R* followed by upregulation of *HL-DR*, *APOE*, *TREM2*, *IFI44L*, *IFITM3*, and *SPP1*. Of note, the T cell co-stimulation molecule CD86 was increased in GBM-associated microglia compared to controls which further shows the potential to activate T cells. On the other hand, *SPP1,* coding for osteopontin, a proinflammatory cytokine involved in neurodegenerative and neuroinflammatory diseases [10,80], was highly expressed in non-diseased microglia from patients older than 50 years compared to younger patients. It is conceivable that the presence of aging-associated microglial clusters in GBM is a reflection of the prevalence of these brain tumors in the senior population. Nonetheless, GBM samples had specific microglial cells that doubled the percentage of IBA1^+^ SPP1^+^ cells of age-matched controls [17]. This becomes relevant since osteopontin induces the recruitment of macrophages through integrin α_v_β_5_ signaling which shows the contribution of microglia to recruit immune cells and shape the TME [81]. In sum, the characterization of microglia in GBM shows a mixture of transcriptional states that includes a population expressing homeostatic core genes, a hypoxia-associated population, an IFN gene signature expressing microglia, and aging-associated microglial clusters, Figure 2. Together, this shows that the preference towards a myeloid phenotype of MDMs or microglia is largely dictated by a specific biological context such as the type of brain cancer they interact with.

## 4. Relationships and Interactions between the Tumor Genetics and Immune Landscape in Gliomas

The concept of genomic profile shaping immune responses in the TME and potential response to therapies is steadily gaining interest in neuro-oncology. Based on the differences in clinical behavior displayed by glioma patients and their distinctive tumor transcriptional profiles [82,83], *IDH1/2* mutations were integrated into the molecular classification of these tumors [84]. In this regard, comprehensive characterizations of the TME of brain metastases, *IDH* wild-type and mutant gliomas were performed [27,53]. These studies have shown that *IDH* wild-type gliomas have a different myeloid cell composition and phenotype compared to those infiltrating *IDH* mutant gliomas. For instance, *IDH* wild-type gliomas were predominantly infiltrated by MDMs in contrast to *IDH* mutant gliomas which were characterized as harboring higher numbers of microglial cells. However, *IDH* wild-type gliomas had more microglial cells than brain metastases. Some of these differences can be attributed to the infiltrating versus focal nature of these malignancies. On the other hand, although not abundant, monocytes represented the main myeloid cell population derived from the blood in *IDH* mutant gliomas, indicating poor recruitment and deficient transition to tissue macrophages as compared to *IDH* wild-type gliomas that are abundantly infiltrated by differentiated MDMs [53]. There are other differences in cellular contents among *IDH* mutant gliomas including astrocytomas having high expression of microglia/macrophages gene signatures whereas oligodendrogliomas have enrichment of neuronal genes [14]. *IDH* mutant gliomas are less infiltrated by T cells and have lower expression of the PD-1 ligand, PD-L1, compared to their wild-type counterpart [85]. The T cell immune suppression in these gliomas is partially attributed to the presence of the oncometabolite (R)-2-hydroxyglutarate generated by the neomorphic activity of the *IDH* mutant enzyme [86]. Although not comparable with the relatively high number of lymphocytes in brain metastases, among *IDH* wild-type GBMs, a few have considerable levels of T cell infiltration which suggests that additional factors contribute to the differences in the composition of the lymphoid compartment among *IDH* wild-type tumors [27].

Although these previous studies have characterized the identity of immune infiltrates in *IDH* wild-type and mutant gliomas, the causal relationship between *IDH* genetic status and tumor-infiltrating immune cells remains a subject for future studies. Furthermore, recent studies characterizing the transcriptional profile of GBM have revealed subtypes associated with specific genetic abnormalities in cancer cells and the immune cellular content. They also provided important insights into the interactions between tumor and immune cells [87]. Compared to proneural and classical subtypes, GBMs classified as mesenchymal contained higher numbers of immune cells including lymphocytes, MDMs and microglia [50,88]. More recently, the depth of genomic deconvolution in GBM has allowed the identification of four cellular states that evolve through time and in accordance with distinct environmental cues. Mesenchymal-like (MES-like), astrocyte-like (AC-like), oligodendrocyte precursor cell-like (OPC-like) and neural progenitor cell-like (NPC-like) states represent these four modifiable oncogenic states described for GBM [89]. Shared biological processes employed during neurodevelopment underpin the expression programs of these cellular states except for the MES-like state which seems to be shaped in part by macrophages and T cell cytotoxic activity [22]. In the context of tumor progression-associated immune changes, innate immune cells contribute to the tumor mesenchymal differentiation predominantly in *IDH* wild-type GBM [68,90]. Upon brain infiltration, macrophages release several cytokines and ligands such as oncostatin M, which binds to the oncostatin M receptor and leukemia inhibitory factor receptor that signals through the signal transducer and activator of transcript 3 pathway to induce the MES-like transcriptional program in glioma cells [22]. This includes the potential of oncostatin M to induce the expression of MHC class I and II by glioma cells which results in increased susceptibility to T cell killing as shown by coculture assays [22]. The influence of macrophages on directing GBM cells towards a particular transcriptional program is also demonstrated by the effect of IFN-γ derived from these immune cells. Secreted IFN-γ induces the expression of immune-related gene signatures such as antigen processing and presentation, response to IFN-γ, response to IFN type I, among others by tumor cells [91]. Overall, these are examples that emphasize the importance of the interaction between tumor cells and the TME to shape the transcriptional and immune identity of glioma cells.

Of note, the mesenchymal signature can be induced without the presence of macrophages and induced by other internal and external cues [90]. On the other hand, considering that the recruitment of MDMs is a consequence of gliomagenesis, the genetic code of brain tumor cells ultimately determines the interaction with these myeloid cells from the beginning of cancer to evolve in a specified direction. For instance, genetic mutations or deletions of *NF1* and *PTEN*, which are common alterations in mesenchymal GBM, appear to be responsible for the recruitment of MDMs and microglia [50]. Upon macrophage infiltration, the dialogue between myeloid cells and glioma cells through ligand-receptor interactions helps both cell types to thrive and synergize in the TME.

## 5. Glioma-Infiltrating Myeloid Cells under Therapy

Glioma-infiltrating myeloid cells are influenced by the standard of care consisting in chemoradiotherapy that GBM patients undergo. Radiotherapy induces substantial transcriptional changes in MDMs and microglia that are displayed when tumors recur. These gene expression changes result in a recurrence signature displayed by both MDMs and microglia [92]. Though a convergence in a transcriptional profile was found upregulated in myeloid cells, radiotherapy modified the myeloid cell abundance represented as an increase in MDMs and a decrease in microglia when tumors recurred in preclinical glioma models [92]. This is consistent with the change observed in the MDMs/microglia ratio between newly diagnosed and recurrent GBM patients [18,92].

GBM patients are often treated with dexamethasone to reduce tumor-associated cerebral edema and treatment-related adverse effects. However, steroid use is associated with worse survival and likely impairs the efficacy of anticancer therapies in GBM [93]. Furthermore, glucocorticoids are known to possess immunosuppressive properties. In fact, they were shown to profoundly affect MDMs and microglia, impairing monocyte tumor infiltration and inducing upregulation of a glucocorticoid gene signature in the myeloid cells isolated from newly diagnosed GBMs [18,94]. This is particularly relevant for studies attempting the use of immunotherapies aimed to exploit anti-tumoral actions of glioma-infiltrating myeloid cells in the upfront setting.

Investigations were conducted to determine whether immune checkpoint blockade impacts the TME of GBM patients in two different temporal contexts of anti-PD-1 therapy administration. One of them, the neoadjuvant administration of PD-1 blockade in recurrent GBM patients showed evidence of increased intratumoral T cells (CD4 and CD8) and early activation of infiltrating CD8^+^ T cells. The T cell receptor repertoire of the intratumoral CD8^+^ T cells overlapped with the peripheral CD8^+^ T cells suggesting that T cell clones generated in the periphery infiltrated tumors as a result of PD-1 blockade [95]. The IFN-γ-stimulated gene signature was also upregulated in GBM samples treated with neoadjuvant PD-1 blockade [96]. This was manifested as an increase in the proportion of myeloid cells expressing IFN-γ stimulated genes and a cluster of PD-L1^+^ monocyte/macrophages [95]. Despite the effects of anti-PD-1 therapy in the TME, GBM remains populated by CD68^+^ myeloid cells expressing the immune checkpoint ligands B7-H3, VISTA and PD-L1. Notably, CD68^+^ macrophage clusters were found to express PD-1 at high levels [97]. Indeed, the therapeutic effect of anti-PD-1 therapy can be attributed partially to the targeting of innate immune cells as shown by increased survival of transgenic glioma models harboring *CD8* gene deletion treated with this immunotherapy [40]. On the other hand, analysis of pre- and post-immunotherapy tumor samples obtained upon recurrence, months after treatment with PD-1 blockade, showed that responder patients to this immunotherapy had decreased *MARCO* expression in recurrent GBM samples [28], a phenomenon not seen in a longitudinal analysis of GBMs treated with chemoradiotherapy [98]. In spatial terms, analysis of pre- and post-immunotherapy *PTEN*-mutated GBM samples showed that macrophages had a higher degree of clustering after treatment with PD-1 blockade [99]. Together, these studies show that immune checkpoint blockade modulates the TME of GBM. However, the abundance of immunosuppressive myeloid cells still represents a challenge for effective immune responses. Nonetheless, neoadjuvant PD-1 blockade shows encouraging results as it induces infiltration of effectors T cells and might increase survival of GBM patients [96].

Even though the results from the Checkmate143 clinical trial did not show survival differences in recurrent GBM patients treated with adjuvant anti-PD-1 therapy [100], recent work from our group suggests that a fraction of these patients have better responses to this immunotherapy assessed by the abundance of p-ERK in tumor regions. GBMs with a robust abundance of p-ERK^+^ cells were associated with a higher number of microglial cells expressing MHC class II compared to tumors with lower p-ERK^+^ cells [101]. Whereas GBM-infiltrating microglia are characterized by upregulating MHC II-associated molecules when compared to control individuals whose expression might be influenced by patient sex [17,102], the presence of these antigen-presenting molecules might determine responses to anti-PD-1 therapy in recurrent GBM patients [101,103].

Considering that the skull bone marrow is a fruitful source of myeloid cells [4], it is important to investigate whether craniotomies performed during the surgical removal of a tumor impact the recruitment and phenotype of macrophages. Along this line, it remains to be determined whether the skull bone marrow contributes to myeloid cells during tumor progression as part of cancer-mediated emergency myelopoiesis. This becomes relevant for immunotherapy since PD-1 inhibition leads to a deviation in the fate of myeloid cell lineages towards an effector phenotype during cancer-driven emergency myelopoiesis that might influence clinical responses to this immunotherapy [38].

Cumulatively, current and experimental therapies influence and modulate the TME of gliomas including MDMs and microglia. It is likely that dynamic interactions between glioma and immune cells ultimately conform to a diverse cellular ecosystem capable of responding to environmental pressures such as anticancer therapies.

## 6. Conclusions

The diverse immune landscapes in GBM are the result of close and dynamic interactions between immune cells and glioma cells. In light of the increasing evidence uncovering differences of the TME in GBM patients, which affect clinical responses to the current standard of care and immunotherapies, it is reasonable to tailor novel treatments based on the immune characteristics of each tumor. Therefore, the dissection of the phenotypical states of glioma-infiltrating myeloid cells and the impact of therapies on them become useful to stratify patients for specific therapies. This is particularly relevant for patients that have marginal or no benefit from alkylating agents that could instead be treated with individualized therapies oriented by the patient’s immune tumor and peripheral landscape [104].

## Data Availability

Not applicable.
